# N-Acetylcysteine Mitigates Renal Fibrosis by Modulating Inflammasome and Gluconeogenic Pathways Under Cardiometabolic Stress

**DOI:** 10.3390/antiox15050636

**Published:** 2026-05-17

**Authors:** Ching-Chun Chen, Hui-Pei Huang, I-Ning Tsai, Huei-Jane Lee, Chau-Jong Wang

**Affiliations:** 1 Institute of Medicine, Chung Shan Medical University, Taichung 40201, Taiwan; sandy985619@gmail.com (C.-C.C.); 0312elise@gmail.com (I.-N.T.); 2 Department of Biochemistry, School of Medicine, Chung Shan Medical University, Taichung 40201, Taiwan; hhpei@csmu.edu.tw; 3 Department of Clinical Laboratory, Chung Shan Medical University Hospital, Taichung 40201, Taiwan; 4 Department of Medical Research, Chung Shan Medical University Hospital, Taichung 40201, Taiwan; 5 Department of Health Industry Technology Management, Chung Shan Medical University, Taichung 40201, Taiwan

**Keywords:** N-acetylcysteine, cardiometabolic stress, renal gluconeogenesis, inflammasome, epithelial–mesenchymal transition, renal fibrosis

## Abstract

Cardio-renal metabolic (CRM) syndrome, characterized by insulin resistance and dyslipidemia, disrupts renal insulin signaling, enhances oxidative stress, and activates inflammasome pathways, ultimately promoting renal fibrosis and kidney dysfunction. Aberrant renal gluconeogenesis has emerged as a critical contributor to tubular injury under cardiometabolic stress; however, its mechanistic linkage to inflammatory and fibrotic remodeling remains incompletely defined. In this study, ApoE^−/−^ mice subjected to streptozotocin administration and a high-fat diet developed pronounced cardiometabolic dysfunction, accompanied by elevated blood urea nitrogen, creatinine, uric acid, and glycated hemoglobin levels, as well as severe renal histopathological alterations. N-Acetylcysteine (NAC) supplementation significantly improved metabolic abnormalities and attenuated tubular dilation, glomerular hypertrophy, and mesangial expansion. Mechanistically, NAC suppressed renal gluconeogenesis by downregulating glucose-6-phosphatase and phosphoenolpyruvate carboxykinase expression and mitigated epithelial–mesenchymal transition by restoring E-cadherin and reducing vimentin expression, thereby limiting fibrotic remodeling. Consistent with in vivo findings, NAC reduced reactive oxygen species production, restored PI3K/Akt-dependent insulin signaling, and inhibited inflammasome activation in NRK-52E renal tubular cells exposed to high glucose and oleic acid, resulting in attenuation of inflammatory signaling and gluconeogenic activity. Collectively, these results demonstrate that NAC mitigates cardiometabolic stress-induced renal injury by modulating inflammasome activation and gluconeogenic reprogramming, highlighting its potential as a mechanistic modulator of renal fibrosis under CRM conditions.

## 1. Introduction

Kidney disease (KD) is a major microvascular complication of metabolic disorders, affecting more than 10% of the global population and contributing substantially to cardiovascular morbidity and mortality [1]. In addition to hyperglycemia-induced renal injury, accumulating evidence indicates that KD arises from multifactorial cardiometabolic disturbances, in which oxidative stress, chronic inflammation, and maladaptive metabolic signaling converge to accelerate renal dysfunction. These systemic insults profoundly alter renal cellular homeostasis and promote progressive fibrotic remodeling [2,3].

Cardio-renal metabolic (CRM) syndrome, characterized by insulin resistance, dyslipidemia, and altered substrate utilization, plays a pivotal role in disrupting renal energy metabolism [4,5]. Cardiometabolic stress influences renal glucose handling through hormonal dysregulation, hemodynamic alterations, and inflammatory mediators [6]. Excessive lipid flux and mitochondrial dysfunction associated with cardiometabolic disorders elevate circulating metabolites and reactive oxygen species (ROS), which interact with renal tubular cells and amplify local metabolic stress. This maladaptive heart–kidney axis promotes aberrant renal gluconeogenic signaling and contributes to systemic glycemic instability. Recent clinical epidemiological studies have shown that CRM syndrome is highly prevalent, affecting up to 71.5% of individuals in large screening cohorts [7,8]. The presence of CRM syndrome is also associated with a markedly increased risk of incident end-stage kidney disease, exceeding a 10-fold elevation in risk. Importantly, robust clinical evidence indicates that concurrent hyperlipidemia and hyperglycemia may synergistically exacerbate renal pathology in humans [9]. Dyslipidemia promotes renal lipotoxicity and macrophage-mediated inflammation, whereas chronic hyperglycemia contributes to glomerular hyperfiltration, oxidative stress, and glomerulosclerosis [10,11,12]. Collectively, these metabolic insults accelerate renal functional decline and structural deterioration, supporting the clinical relevance of CRM syndrome in the progression of kidney disease.

The kidney is a key regulator of systemic glucose homeostasis through gluconeogenesis, a process primarily localized in proximal tubular epithelial cells [13]. Renal gluconeogenesis is tightly controlled by insulin signaling via the phosphoinositide 3-kinase (PI3K)/Akt pathway and its downstream transcription factor forkhead box protein O1 (FOXO1). Under diabetic and cardiometabolic conditions, impaired insulin signaling and inflammatory activation result in dysregulated FOXO1 activity and aberrant upregulation of gluconeogenic enzymes, including phosphoenolpyruvate carboxykinase (PEPCK) and glucose-6-phosphatase (G6Pase) [14]. Sustained activation of renal gluconeogenesis exacerbates hyperglycemia, increases oxidative burden, and imposes metabolic stress on tubular cells, thereby facilitating fibrotic progression. Chronic cardiometabolic stress is closely linked to activation of the Nucleotide-binding oligomerization domain-like receptor (NLR) family pyrin domain containing 3 (NLRP3) inflammasome, a multiprotein complex that mediates caspase-1–dependent maturation of interleukin-1β (IL-1β) [15,16]. In patients with CKD and DKD, prolonged glucolipotoxicity promotes ectopic glucolipid accumulation in renal cells, thereby inducing substantial oxidative stress and the generation of lipid peroxides [17,18]. These metabolic stressors may act as danger-associated molecular patterns that prime and promote NLRP3 inflammasome assembly through the NF-κB signaling pathway [17,19,20]. Subsequent IL-1β release not only contributes to pyroptotic cell death but also disrupts PI3K/Akt-mediated insulin signaling, thereby establishing a vicious cycle of inflammation and metabolic dysregulation. Collectively, these pathological processes accelerate glomerulosclerosis and contribute to progressive and irreversible renal failure [20,21].

In renal tubular epithelial cells, persistent oxidative and inflammatory stimuli activate transforming growth factor-β (TGF-β) signaling and initiate epithelial–mesenchymal transition (EMT), characterized by loss of epithelial markers such as E-cadherin and induction of mesenchymal markers including vimentin [22]. These phenotypic changes promote extracellular matrix accumulation and drive renal fibrotic remodeling [23,24].

Apolipoprotein E–deficient (ApoE^−/−^) mice exhibit spontaneous dyslipidemia and have been widely employed as a model of cardiometabolic dysfunction and advanced atherosclerosis [25,26,27]. A high-fat diet (HFD) exacerbates metabolic disturbances in this model, leading to severe vascular oxidative stress, endothelial dysfunction, elevated inflammatory markers, increased lipid accumulation, and enhanced plaque burden with spontaneous arterial rupture [28,29]. To simulate combined metabolic and glycemic stress, ApoE^−/−^ mice are fed an HFD to induce aortic atherosclerosis and subsequently administered streptozotocin to induce hyperglycemia [30]. Recent evidence further demonstrates that superimposing diabetic conditions on the ApoE^−/−^ background significantly accelerates cardiovascular pathologies [31]. Therefore, this integrated model provides a relevant experimental platform to examine how systemic cardiometabolic stress alters renal gluconeogenic regulation and accelerates fibrotic kidney injury.

N-Acetylcysteine (NAC) is a clinically used antioxidant with established redox-modulating and anti-inflammatory properties [32]. Beyond its role as a glutathione precursor, NAC has been shown to suppress pro-inflammatory cytokine production and modulate NLRP3 inflammasome activity [33]. Clinical and experimental evidence suggests that NAC may attenuate oxidative stress–related renal injury [34,35]. In CKD, NAC plays an important role in replenishing the markedly depleted endogenous glutathione pool. However, its relatively low oral bioavailability, largely attributable to extensive first-pass metabolism, requires careful dose optimization to support effective clinical translation. However, its potential to simultaneously regulate inflammasome activation and gluconeogenic reprogramming under cardiometabolic stress remains poorly defined. Therefore, the present study employs combined in vivo and in vitro models to investigate the role of cardiometabolic stress in renal gluconeogenic dysregulation and fibrotic remodeling, and to evaluate whether NAC mitigates renal fibrosis by modulating inflammasome and gluconeogenic pathways.

## 2. Materials and Methods

### 2.1. Animal Experiments

Male apolipoprotein E-deficient (ApoE^−/−^) mice on a C57BL/6J background, 7 weeks of age and weighing 18–20 g, were obtained from the National Laboratory Animal Center (Taipei, Taiwan). Animals were housed under controlled environmental conditions (22 ± 2 °C; 12 h light/12 h dark cycle). All procedures were reviewed and approved by the Institutional Animal Care and Use Committee of Chung Shan Medical University (CSMU; IACUC approval no. 112053, Date: 14 September 2023) and conducted in accordance with the Taiwan Animal Protection Act (Law No. 10500042801, enacted 4 November 1998; amended 18 May 2016) and the institutional animal care guidelines (R&D Department, 20 June 2014). ApoE^−/−^ mice were employed as a model of cardiometabolic dysfunction, whereas age-matched C57BL/6J mice served as normal controls. Diabetes was induced following the Animal Models of Diabetic Complications Consortium (AMDCC) protocol. After a 12 h fast, mice received intraperitoneal injections of streptozotocin (STZ; 50 mg/kg; Sigma-Aldrich, St. Louis, MO, USA) freshly prepared in 0.1 M citrate buffer (pH 4.5) once daily for five consecutive days. All mice were fed a standard chow diet during the 5-day streptozotocin. One week after the final injection, blood glucose levels were measured via tail vein sampling, and mice with glucose concentrations exceeding 200 mg/dL were classified as diabetic and enrolled in subsequent experiments. The animals were randomly assigned to experimental groups (*n* = 5) and given a standard diet or HFD with NAC treatment for the next 8 weeks.

1. Control, C: Age-matched C57BL/6J mice fed a standard chow diet (5010; 24.6% protein, 6.1% ash, 5.0% fat, 4.2% crude fiber) for 8 weeks.

2. Normal control, NC: ApoE^−/−^ mice maintained on a standard chow diet for 8 weeks.

3. STZ: ApoE^−/−^ mice administered STZ and subsequently fed a standard chow diet for 8 weeks.

4. STZ+HFD: ApoE^−/−^ mice administered STZ and fed an HFD (17% lard, 1.2% cholesterol, and 0.2% sodium cholate hydrate) for 8 weeks.

5. STZ + HFD + NAC: ApoE^−/−^ mice administered STZ, HFD, and treated with NAC (200 mg/kg/day, oral gavage, Sigma-Aldrich, St. Louis, MO, USA) for 8 weeks.

Fresh diets were provided daily, and residual feed was weighed to determine daily food intake. The blood glucose was monitored biweekly. At the end of the experimental period, mice were deeply anesthetized with inhaled isoflurane. After confirmation of the absence of pain reflexes, blood was collected by cardiac puncture, followed by euthanasia through exsanguination, in accordance with humane endpoint criteria. Blood samples were collected via cardiac puncture for biochemical analysis. Kidneys were excised, weighed, and processed for histopathological and molecular analysis.

### 2.2. Renal Function and Biochemical Analyses

Blood samples collected at sacrifice were centrifuged at 3000× *g* for 10 min at 4 °C to obtain plasma. Plasma urea nitrogen (BUN; UR107), creatinine (CR510), and uric acid (UA230) were determined using enzymatic colorimetric assays with commercial kits (Randox Laboratories, Antrim, UK). Glycated hemoglobin (HbA1c) was measured using a kit from Fortress Diagnostics (Antrim, UK), and plasma insulin concentrations were quantified with a mouse insulin ELISA kit (Mercodia AB, Uppsala, Sweden).

### 2.3. Histopathological Analysis of Kidney

Kidney specimens were fixed in 10% neutral-buffered formalin, dehydrated, and embedded in paraffin. Sections (4 μm) were deparaffinized and stained with hematoxylin and eosin (H&E), Masson’s trichrome, or periodic acid–Schiff (PAS) to evaluate tissue morphology, fibrosis, and basement membrane integrity, respectively.

H&E staining: Sections were stained in hematoxylin for 25 s, rinsed, counterstained with eosin, dehydrated through graded alcohols, cleared in xylene, and mounted.

Masson’s trichrome staining: Sections were fixed in Bouin’s solution, rinsed, stained with Weigert’s iron hematoxylin for 10 min, followed by Biebrich scarlet–acid fuchsin for 7 min, differentiation in phosphomolybdic–phosphotungstic acid, staining with aniline blue, and final dehydration in graded alcohols.

PAS staining: Sections were oxidized, treated with Schiff’s reagent to produce magenta coloration, and counterstained with hematoxylin.

Slides were examined under a TissueFAXS PLUS tissue cytometer (TissueGnostics, Vienna, Austria), and histological changes in glomerular and tubular structures were evaluated. All Representative images were selected by blinded investigators from multiple random fields (*n* = 3) to best reflect the mean quantitative results of each group. Data are expressed as fold changes relative to the control.

### 2.4. Renal Cell Culture

Rat renal proximal tubular epithelial cells (NRK-52E; Bioresource Collection and Research Center, Hsinchu, Taiwan) were cultured in Dulbecco’s Modified Eagle’s Medium (DMEM; Gibco, Gaithersburg, MD, USA) supplemented with 5% fetal bovine serum, 1% penicillin–streptomycin, 4 mM L-glutamine, 4.5 g/L glucose, and 1.5 g/L sodium bicarbonate. Cells were maintained in a humidified atmosphere at 37 °C with 5% CO_2_ and 95% air.

### 2.5. MTT Assay

NRK-52E cells were seeded in 24-well plates (1 × 10^5^ cells/mL) and allowed to attach for 16 h prior to treatment. Cells were exposed to high glucose (0–50 mM), oleic acid (0–320 μM), or NAC (250–1000 μM) for 24 h. After treatment, the medium was replaced with MTT solution (0.5 μg/mL) and incubated for 3 h at 37 °C. Formazan crystals were dissolved in isopropanol, and absorbance was measured at 563 nm using a spectrophotometer (U2001, Hitachi, Tokyo, Japan). Cell viability was expressed as a percentage of the untreated control. Data represent the mean of three independent biological replicates.

### 2.6. Western Blot Analysis

After 24 h of treatment, cells were lysed in RIPA buffer and centrifuged at 14,000× *g* for 15 min at 4 °C. Protein concentrations were determined using the BCA assay. Equal amounts of protein (50 µg) were resolved by SDS–PAGE and transferred to PVDF membranes (Pall Life Sciences, NY, USA). Membranes were blocked with BlockPRO^TM^ protein-free blocking buffer (Visual Protein BP01-1L, Taipei City, Taiwan) for 30 min and incubated overnight at 4 °C with primary antibodies. After washing, membranes were incubated with horseradish peroxidase–conjugated secondary antibodies (GE Healthcare, Little Chalfont, UK) for 1 h. Protein bands were visualized using enhanced chemiluminescence (ECL; Fujifilm LAS-4000, Tokyo, Japan) and quantified with Multi Gauge v2.2 software (Fujifilm, Tokyo, Japan). Target protein levels were normalized to β-actin, and band quantification was performed by blinded investigators.

### 2.7. Immunohistochemistry Analysis (IHC)

After tissue deparaffinization, the sections were rehydrated through a graded ethanol series. Antigen retrieval was conducted by immersing the sections in citric acid buffer and heating in water bath for 15 min. After cooling to room temperature, the sections were washed with TBST buffer. Endogenous peroxidase activity was blocked by incubation with a DAB inhibitor at room temperature for 10 min. Sections were washed and blocked with TBST containing 10% fetal bovine serum (FBS) for 1 h to reduce nonspecific binding. The sections were then incubated with primary antibodies against G6Pase (1:250; Abcam, UK; ab319053), PEPCK (1:250; Santa Cruz, CA, USA; sc-271029), E-cadherin (1:250; Santa Cruz, USA; sc-7870), or Vimentin (1:250; Santa Cruz, USA; sc-6260) at 37 °C for 1–2 h. After primary antibody incubation, the sections were washed with TBST and incubated with HRP multimer at room temperature for 50 min. Chromogenic detection was carried out using a DAB working solution prepared at a 1:1 ratio of DAB chromogen to DAB H_2_O_2_ at room temperature. The reaction was terminated by washing, and the sections were counterstained with hematoxylin for 8–10 s. The quantification of protein level was performed by examining ten randomly selected cells under a light microscope (Zeiss Axio Imager.Z2, Germany). Image acquisition and intensity quantification were performed by blinded investigators. Results are expressed as fold changes relative to the control.

### 2.8. Immunocytochemistry (ICC) Analysis In Vitro and In Vivo

NRK-52E cells (1 × 10^5^ cells/well) were seeded in 24-well plates and treated overnight with high glucose (25 mM), oleic acid (80 μM), or NAC (500 μM). Cells were washed with phosphate-buffered saline (PBS), fixed with 4% paraformaldehyde for 10 min, and permeabilized with 0.1% Triton X-100 for 5 min. Kidney sections were deparaffinized, rehydrated through graded ethanol, and subjected to antigen retrieval in 0.01 M citrate buffer (pH 6.0) at 100 °C for 15 min, followed by permeabilization with 0.1% Triton X-100 for 10 min. After blocking with 1% bovine serum albumin for 30–60 min, cells and tissue sections were incubated overnight at 4 °C with primary antibodies against G6Pase (1:250; Abcam, ab319053), PEPCK (1:250; Santa Cruz, sc-271029), E-cadherin (1:200; Santa Cruz, sc-7870), vimentin (1:250; Santa Cruz, sc-6260), or IL-1β (1:250; Abcam, ab283818). Following PBS washes, samples were incubated with Alexa Fluor 488- or 584-conjugated secondary antibodies for 1 h at room temperature. F-actin was visualized using phalloidin, and nuclei were counterstained with 4′,6′-diamidino-2-phenylindole (DAPI). Fluorescence images were acquired using a BX53 fluorescence microscope (Olympus, Tokyo, Japan). Image acquisition and intensity quantification were performed by blinded investigators. Results are expressed as fold changes relative to the control.

### 2.9. Determination of Reactive Oxygen Species (ROS)

Reactive oxygen species (ROS) levels were measured using 2′,7′-dichlorodihydrofluorescein diacetate (H_2_DCFDA, also known as DCFH-DA). Owing to its cell-permeable property, H_2_DCFDA is hydrolyzed by intracellular esterases to non-fluorescent 2′,7′-dichlorodihydrofluorescein (HDCF), which is subsequently oxidized by ROS to form the highly fluorescent compound 2′,7′-dichlorofluorescein (DCF). The intracellular fluorescence intensity of DCF was quantified to assess ROS levels. This assay was performed in NAC-treated NRK-52E cells. After treatment with NAC (500 μM), cells were incubated with 10 μM H_2_DCFDA for 30 min. Fluorescence intensity was analyzed using a BD FACSCanto™ II flow cytometer, and data are presented as the mean ± standard deviation (SD) of three independent biological replicates.

### 2.10. Molecular Docking

Molecular docking was performed to evaluate the interactions between NAC and G6Pase and PEPCK. Protein structures were obtained from the AlphaFold Protein Structure Database (PBD codes, G6Pase, 9JTN; PEPCK, 1KHB), and the NAC structure was retrieved from PubChem (CID 12035). Protein preparation, including removal of water molecules, elimination of nonessential residues, and addition of hydrogen atoms and atomic charges, was performed using PyMOL (v3.0.4, http://www.pymol.org/, Schrödinger Inc., New York, NY, USA). The binding affinities were evaluated based on predicted binding energies. Docking poses were analyzed to identify potential interaction residues and binding regions, including the M0–M2 docking sites in G6Pase and M1 in PEPCK. Structural interactions were visualized using PyMOL.

### 2.11. Statistical Analysis

All statistical analyses were performed using GraphPad Prism software (version 8.0.2; GraphPad Software, San Diego, CA, USA). Data are presented as the mean ± SD. Differences among multiple groups were assessed using one-way analysis of variance (ANOVA), followed by Bonferroni’s post hoc test. For all analyses, n represents the number of independent biological replicates. A two-tailed *p*-value < 0.05 was considered statistically significant.

## 3. Results

### 3.1. NAC Improves Renal Function in ApoE^−/−^ Mice Under Cardiometabolic Stress

**Figure 1:**
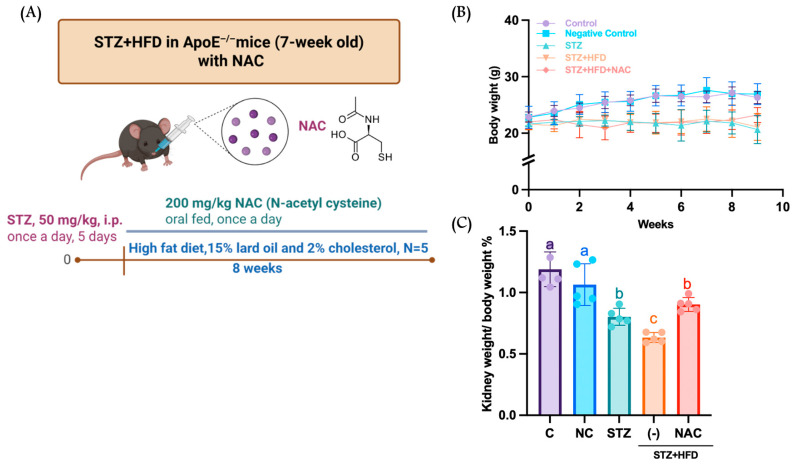
Effects of NAC on body weight and kidney weight change in HFD-fed C57BL6 and ApoE^−/−^ mice. C57BL6 and ApoE^−/−^ mice were fed with normal diet, high fat diet and NAC. The (**A**) body weight and (**B**) kidney weight change in mice were measured once a week. (**C**) C57BL6 mice were fed with normal diet; NC, ApoE^−/−^ mice were fed with normal diet; STZ, ApoE^−/−^ mice were fed with normal diet; STZ+HFD, ApoE^−/−^ mice were fed with HFD; STZ+HFD +NAC, ApoE^−/−^ mice were fed with HFD and NAC. Quantification was performed using one-way ANOVA followed by Bonferroni’s post hoc test. All data are shown as mean ± SD. Individual data points are overlaid as dots on the bars, each representing an independent experiment (*n* = 5). Groups not sharing a common letter are significantly different (*p* < 0.05).

As shown in Figure 1, ApoE^−/−^ mice administered STZ exhibited a 22.3% reduction in body weight compared with the NC group at the end of the experimental period. NAC supplementation significantly attenuated this weight loss, resulting in an approximately 11.5% increase in body weight relative to the STZ group. Moreover, kidney weight in the STZ group was reduced by approximately 11.2% compared with the NC group, whereas kidney weight in NAC-treated mice did not differ significantly from that of the untreated NC group. Relative to ApoE^−/−^ mice, the STZ group showed significant increases in uric acid and glycated hemoglobin levels. Additionally, the STZ+HFD group exhibited further elevations in BUN, creatinine, uric acid, glycated hemoglobin, and insulin levels compared with the STZ group (Table 1), indicating exacerbated renal impairment. NAC treatment for 8 weeks markedly improved all measured biochemical parameters in the STZ+HFD+NAC group compared with the STZ+HFD group, demonstrating that NAC effectively ameliorates cardiometabolic dysfunction–associated renal injury in this model.

### 3.2. NAC Improves Renal Histological Changes Under Cardiometabolic Stress

**Figure 2:**
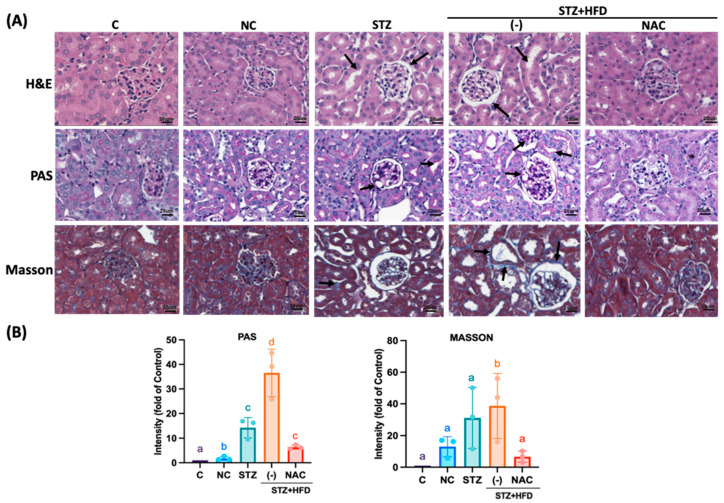
NAC reduced tubular dilation, mesangial cell proliferation, and matrix expansion in STZ+HFD ApoE^−/−^ mice. Kidney sections were embedded in paraffin and stained with (**A**), hematoxylin and eosin. Light microscopy photos of kidney sections were shown. Bars: 20 µm. PAS and Masson stain were counted using the ipwin32 software (versions v11.1). Arrows indicate the renal tubule. (**B**), Quantification was performed as aforementioned (*n* = 3). Groups not sharing a common letter are significantly different (*p* < 0.05). While the total animal cohort consisted of 5 mice per group (*n* = 5) for physiological measurements, the individual data points overlaid on the bar graphs represent three randomly selected independent biological replicates (*n* = 3) utilized specifically for this ex vivo histological and molecular analysis.

Results demonstrated that ApoE^−/−^ mice administered HFD and STZ developed pronounced renal injury (Figure 2), characterized by marked tubular dilation, glomerular hypertrophy, mesangial expansion, and increased collagen deposition, consistent with aggravated renal damage. In contrast, STZ+HFD+NAC group mice exhibited substantially attenuated histopathological alterations. NAC supplementation reduced tubular dilation, diminished mesangial matrix accumulation, and limited interstitial fibrosis, as evidenced by a decreased proportion of Masson’s trichrome–positive staining.

### 3.3. NAC Reduces Renal Gluconeogenesis and Fibrosis in STZ+HFD ApoE^−/−^ Mice

**Figure 3:**
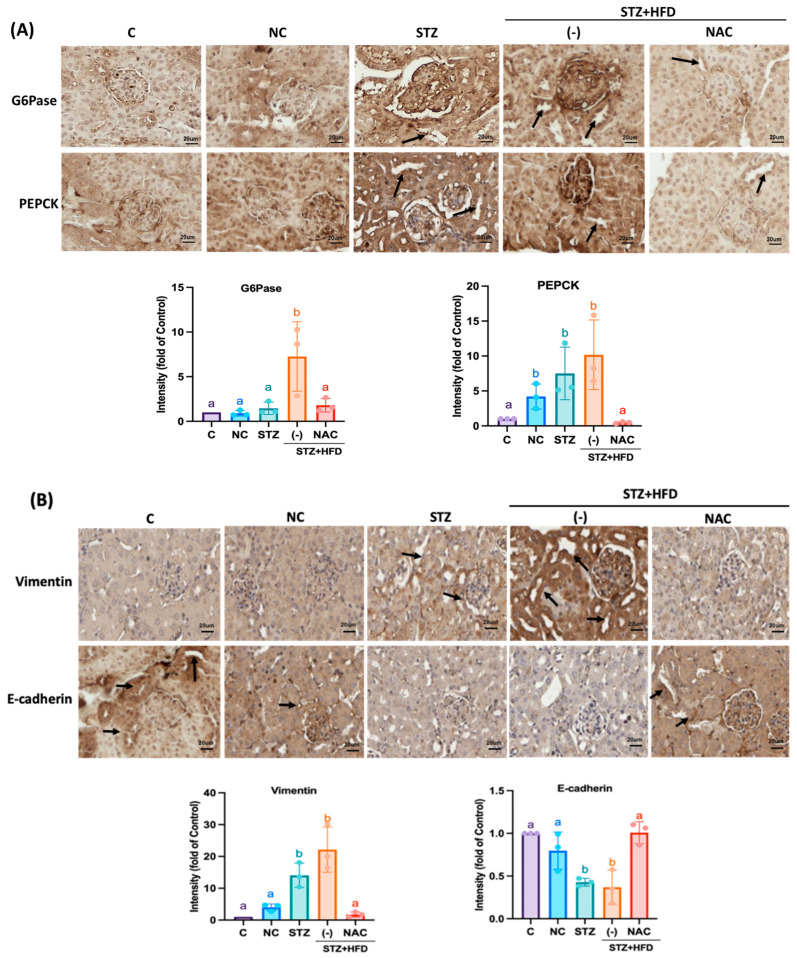
NAC reduces gluconeogenesis and EMT protein in the proximal tubule of STZ+HFD ApoE^−/−^ mice. Renal (**A**) G6Pase and PEPCK and (**B**) E-cadherin and Vimentin in the NAC group were displayed. (Bars: 20 mm.) As a C group, C57BL6 mice were fed a normal diet; NC and STZ group were fed a normal diet; STZ+HFD were fed HFD diet, STZ+HFD+NAC group were fed HFD and treatment NAC, respectively. Quantification of protein expression was performed using one-way ANOVA followed by Bonferroni’s post hoc test. Data are shown as mean ± SD. Individual data points are overlaid as dots on the bars, each representing an independent experiment (*n* = 3). Groups not sharing a common letter are significantly different (*p* < 0.05). All Representative images were selected by blinded investigators from multiple random fields (*n* = 3) to best reflect the mean quantitative results of each group.

**Figure 4:**
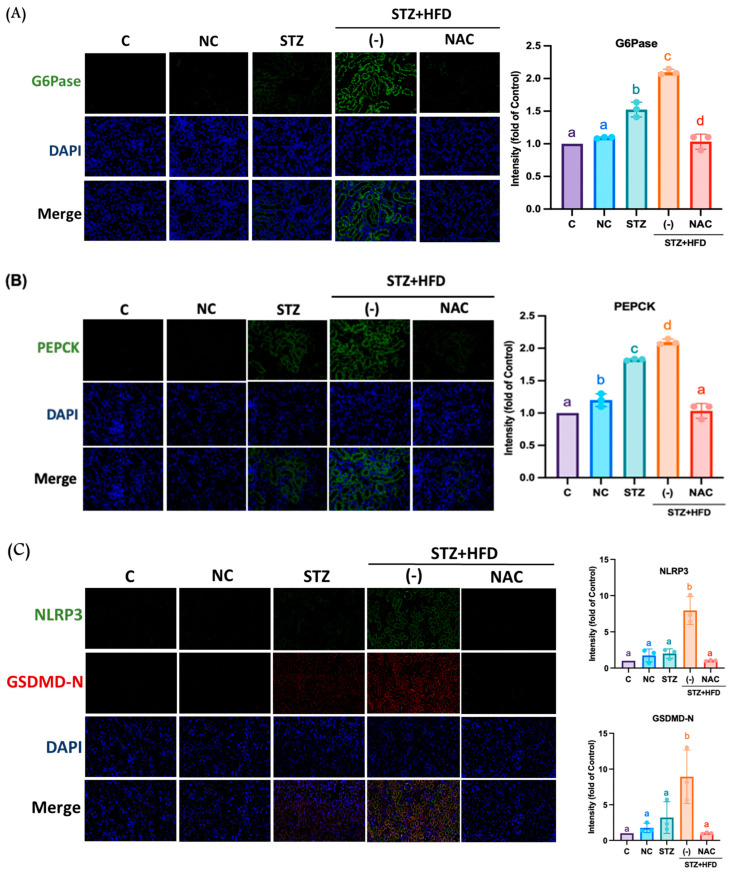

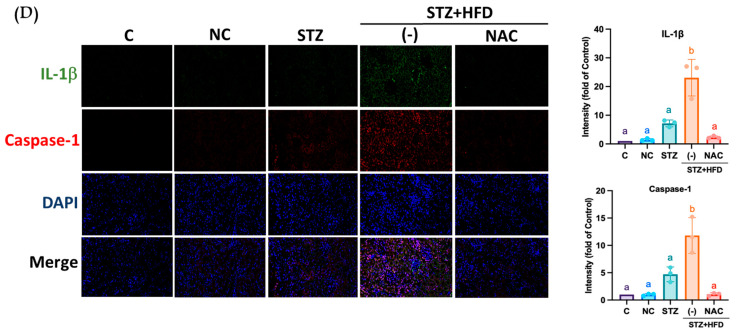
NAC reduces the fluorescent expression of gluconeogenesis and inflammasome-associated protein on the STZ+HFD ApoE^−/−^ mice model. Shown is the fluorescence expression of (**A**), G6Pase; (**B**), PEPCK; (**C**), NLRP3 and GSDMD; (**D**), IL-1β and Caspase-1 in the kidneys of the NAC group (200× magnification). As a C group, C57BL6 mice were fed a normal diet; NC and STZ group were fed a normal diet; STZ+HFD were fed HFD, STZ+HFD+NAC group were fed HFD and treatment NAC, respectively. Protein fluorescence was counted using the ipwin32 software. Quantification was performed using one-way ANOVA followed by Bonferroni’s post hoc test. Data are shown as mean ± SD. Individual data points are overlaid as dots on the bars, each representing an independent experiment (*n* = 3). Groups not sharing a common letter are significantly different (*p* < 0.05). All Representative images were selected by blinded investigators from multiple random fields (*n* = 3) to best reflect the mean quantitative results of each group.

As shown in Figure 3A, the STZ+HFD group exhibited markedly elevated expression of PEPCK, a key rate-limiting enzyme in gluconeogenesis, as well as G6Pase, which catalyzes the terminal step of the gluconeogenic pathway, 8.11 and 10-fold compared with the NC group. NAC treatment significantly reduced the expression of both enzymes by 2.3 and 0.8-fold, respectively. EMT, a process known to promote extracellular matrix production and fibrosis, was also dysregulated in the STZ+HFD group, as evidenced by 22.3-fold increased Vimentin expression and 0.4-fold decreased E-cadherin levels (Figure 3B). NAC administration reversed these alterations by downregulating vimentin expression to 1.5-fold and restoring E-cadherin expression to 1-fold. Consistently, immunofluorescence analysis (Figure 4A,B) showed a significant increase in marked cytoplasmic of PEPCK and G6Pase in the STZ+HFD group by 2.3-fold and 2.15-fold, whereas NAC-treated mice displayed expression levels comparable to controls. The NLRP3 inflammasome is a key multiprotein complex that mediates upstream inflammatory signaling and contributes to renal fibrogenesis. Figure 4C,D shows that the fluorescence expression of inflammasome-related proteins such as NLRP3, GSDMD, IL-1β, and caspase-1 was significantly increased by 7.9, 8.9, 23 and 11.8-fold in the STZ+HFD-induced group, but was effectively reduced by 1.02, 1.03, 2.2, and 1.05-fold in the NAC-treated group.

### 3.4. NAC Suppresses OA+HG-Induced Inflammation and Renal Gluconeogenesis in Cell Models

**Figure 5:**
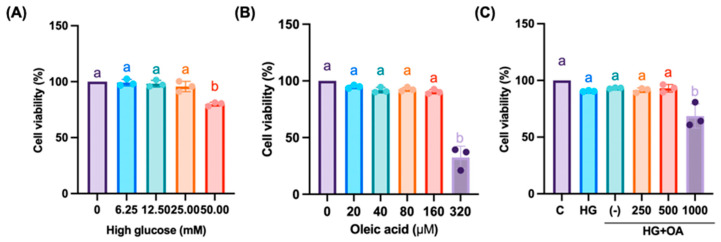
Effect of NAC on glycolipotoxicity induced in NRK-52E proximal tubule cells. NRK-52E cells were treated with indicated (**A**), concentration of high glucose (0, 6.25, 12.5, 25, 50 mM); (**B**), oleic acid (0, 20, 40, 80, 160, 320 μM) only; (**C**), co-treated with high glucose (25 mM), oleic acid (80 μM), and NAC (0, 250, 500, 1000 μM) for 24 h. Quantification of cell viability was performed using one-way ANOVA followed by Bonferroni’s post hoc test. Data are shown as mean ± SD. Individual data points are overlaid as dots on the bars, each representing an independent experiment (*n* = 3). Groups not sharing a common letter are significantly different (*p* < 0.05).

**Figure 6:**
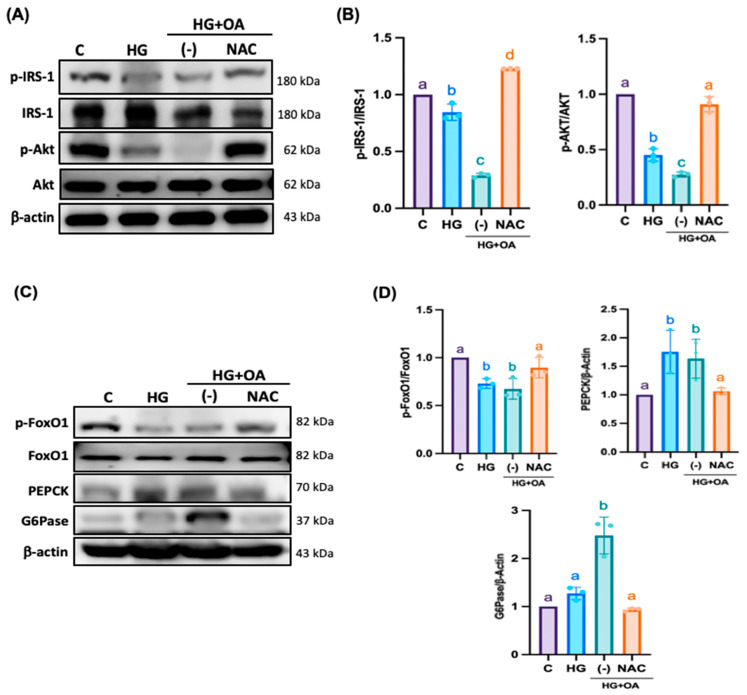

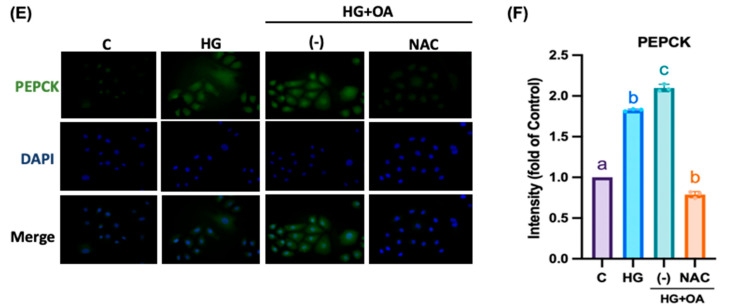
NAC restores IRS-1/Akt signaling and suppresses gluconeogenic protein expression in HG + OA–treated NRK-52E cells. NRK-52E proximal tubular epithelial cells were treated with HG (25 mM), OA (80 μM) and NAC (500 μM) for 24 h. (**A**–**D**) Phosphorylated IRS-1, phosphorylated Akt, FoxO1, G6Pase, and PEPCK were examined by Western blotting. (**E**,**F**) Immunofluorescence staining was performed to visualize PEPCK expression, and representative images and immunoblots are presented. Confocal scanning microscopy showing PEPCK fluorescence on NRK-52E cells (green: PEPCK, blue: DAPI, 200× magnification). Protein fluorescence was counted using the ipwin32 software. Quantification was performed using one-way ANOVA followed by Bonferroni’s post hoc test. Data are shown as mean ± SD. Individual data points are overlaid as dots on the bars, each representing an independent experiment (*n* = 3). Groups not sharing a common letter are significantly different (*p* < 0.05). All Representative images were selected by blinded investigators from multiple random fields (*n* = 3) to best reflect the mean quantitative results of each group.

To elucidate the mechanisms by which NAC influences inflammasome activation and gluconeogenesis, NRK-52E cells were exposed to high glucose (HG) and oleic acid (OA). Cytotoxicity assays were first performed using increasing concentrations of HG (0–50 mM) and OA (0–320 μM). As shown in Figure 5A,B, HG concentrations exceeding 25 mM and OA concentrations above 80 μM significantly reduced NRK-52E cell viability. Thus, 25 mM HG and 80 μM OA were selected as non-cytotoxic conditions for subsequent experiments. NAC (0–1000 μM) did not affect cell viability after 24 h, and 500 μM was chosen for further analyses (Figure 5C). Insulin receptor substrate-1 (IRS-1) and Akt are key mediators of insulin signaling. In HG-treated cells, co-treatment with OA markedly decreased phosphorylated IRS-1 and Akt to 0.20-fold and 0.55-fold of control levels, respectively. NAC treatment effectively restored phosphorylation of both proteins (Figure 6A,B). FOXO1 phosphorylation via the PI3K/Akt axis suppresses gluconeogenic enzymes. OA exposure decreased p-FOXO1 to 0.67-fold of control and G6Pase (2.5-fold), and PEPCK (4.0-fold) compared with controls (*p* < 0.05), whereas NAC significantly restored p-FOXO1 (0.92-fold) and reduced G6Pase and PEPCK protein levels. Immunofluorescence staining confirmed attenuation of PEPCK expression following NAC treatment (Figure 6C–F). Collectively, these findings indicate that NAC alleviates lipotoxicity- and hyperglycemia- induced dysregulation of renal gluconeogenesis and restores insulin-mediated signaling in NRK-52E cells.

### 3.5. NAC Inhibits Inflammatory Signaling and Fibrosis Pathways in NRK-52E Cells

**Figure 7:**
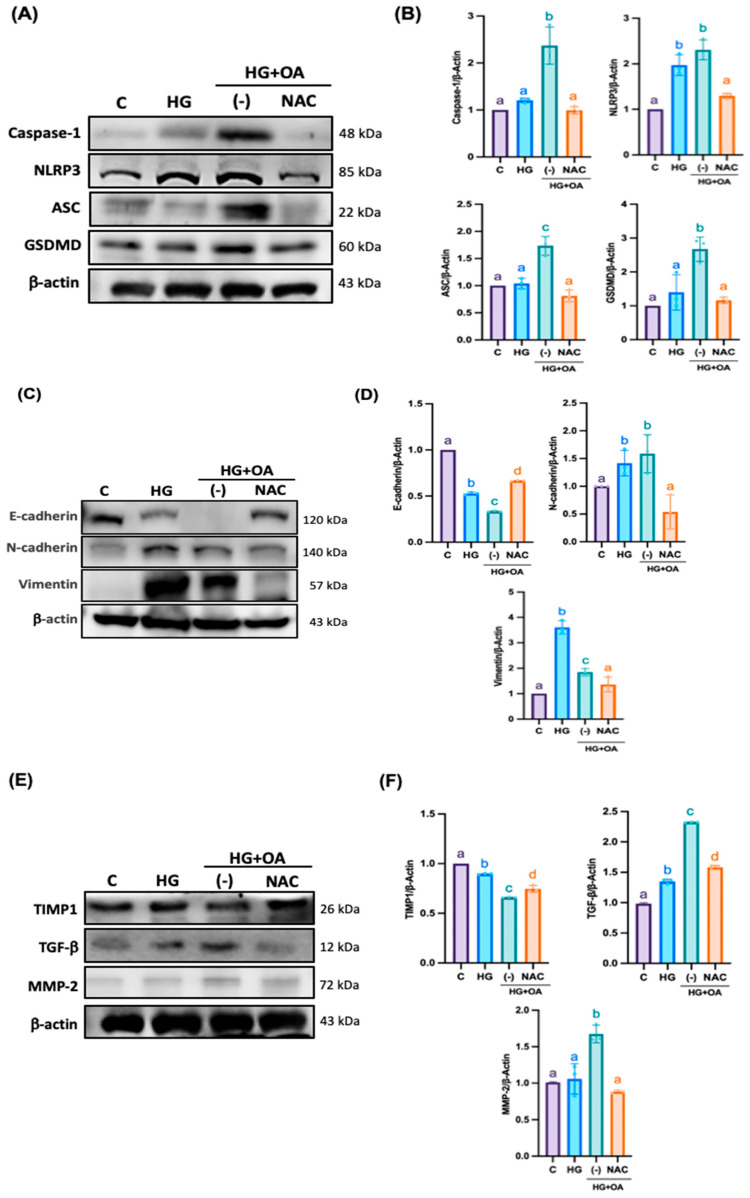
NAC suppresses inflammasome activation and modulates EMT-associated fibrotic signaling in NRK-52E cells. NRK-52E cells were treated with high glucose (25 mM), oleic acid (80 μM), and NAC (500 μM) for 24 h. (**A**,**B**) Inflammasome complex markers included Caspase-1, NLRP3, ASC, and GSDMD, while (**C**,**D**) EMT markers included E-cadherin, N-cadherin, and vimentin, and (**E**,**F**) ECM markers included TGF-β, TIMP-1, and MMP-2, all of which were determined by Western blot analysis. Representative Western blot images and corresponding quantitative densitometric analyses were normalized to β-actin. Statistical comparisons were performed using one-way ANOVA, followed by Bonferroni’s post hoc test. Data are presented as the mean ± SD. Individual data points are superimposed on the bar graphs, with each dot representing an independent experiment (*n* = 3). Groups not sharing a common letter are significantly different (*p* < 0.05).

**Figure 8:**
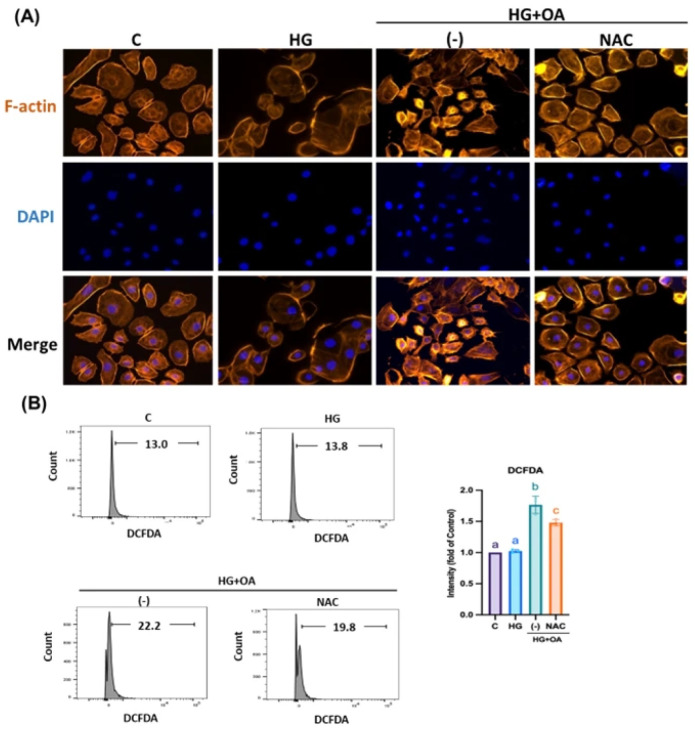
NAC restores cytoskeletal integrity and reduces oxidative stress activation in HG + OA–stimulated NRK-52E cells. (**A**) Immunofluorescence staining of F-actin was performed to assess cytoskeletal morphology. Confocal scanning microscopy showing F-actin fluorescence on NRK-52E cells (red: F-actin, blue: DAPI, 200× magnification). (**B**) Intracellular ROS levels were quantified by flow cytometry. Statistical comparisons were performed using one-way ANOVA, followed by Bonferroni’s post hoc test. Data are presented as the mean ± SD. Individual data points are superimposed on the bar graphs, with each dot representing an independent experiment (*n* = 3). Groups not sharing a common letter are significantly different (*p* < 0.05). All Representative images were selected by blinded investigators from multiple random fields (*n* = 3) to best reflect the mean quantitative results of each group.

**Figure 9:**
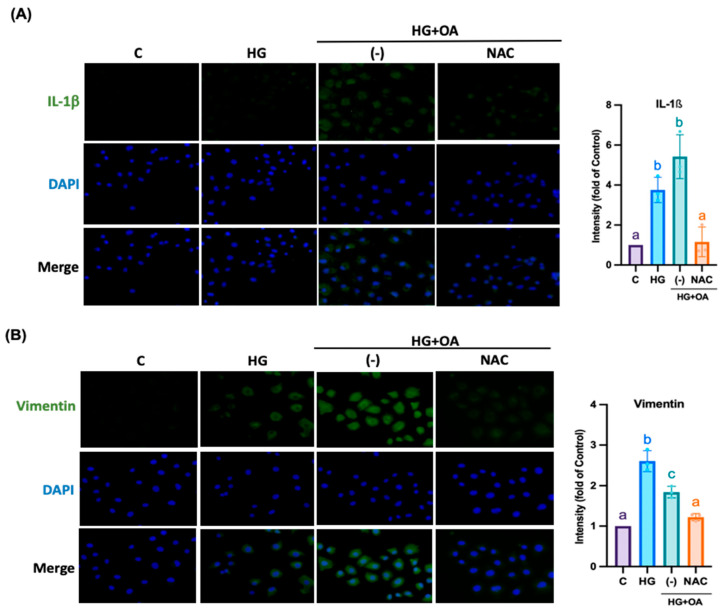
NAC reduced Epithelial–Mesenchymal Transition marker-related protein expression in NRK-52E cells. NRK-52E cells were treated with high glucose (25 mM), oleic acid (80 μM), and NAC (500 μM) for 24 h. Confocal scanning microscopy showing (**A**), IL-1β and (**B**), Vimentin fluorescence on NRK-52E cells (green: IL-1β and Vimentin, blue: DAPI, 200× magnification). Protein fluorescence was performed using the ipwin32 software. Statistical comparisons were performed using one-way ANOVA, followed by Bonferroni’s post hoc test. Data are presented as the mean ± SD. Individual data points are superimposed on the bar graphs, with each dot representing an independent experiment (*n* = 3). Groups not sharing a common letter are significantly different (*p* < 0.05). All Representative images were selected by blinded investigators from multiple random fields (*n* = 3) to best reflect the mean quantitative results of each group.

Furthermore, inflammasomes promote EMT and accelerate the pathological process of kidney injury. As shown in Figure 7A,B, OA-treated NRK-52E cells exhibited markedly elevated levels of Caspase-1, NLRP3, ASC and gasdermin D (GSDMD) by 2.4, 2.45, 1.78 and 2.81-fold compared with controls, indicating robust inflammasome activation. NAC treatment significantly reduced the expression of all three proteins to 1-fold, 1.25-fold, 0.85-fold, and 1.1-fold, restoring them to near-control levels. EMT, a critical driver of ECM accumulation and renal fibrosis, was also markedly induced by OA exposure. OA treatment increased the expression of profibrotic proteins N-cadherin, vimentin, TGF-β, and MMP-2 to 1.55, 2, 2.3 and 1.7-fold, while decreasing epithelial markers such as E-cadherin to 0.35-fold and the antifibrotic inhibitor TIMP1 to 0.7-fold. NAC treatment reversed these alterations by suppressing EMT-associated proteins and restoring E-cadherin and TIMP1 expression by 0.75-fold and 0.85-fold (Figure 7C–F). NRK-52E cells treated with high glucose and oleic acid exhibited severe cytoskeletal disruption, characterized by fragmented and disorganized F-actin filaments (Figure 8A). High glucose and OA increased cellular oxidative stress by 0.61-fold, confirming inflammasome activation (Figure 8B). NAC co-treatment restored F-actin organization and reduced oxidative stress levels by 0.2-fold, respectively. These findings suggest that NAC may attenuate oxidative stress; however, broader redox profiling is required to confirm its comprehensive antioxidant effects in renal tissue. Furthermore, in the HG + OA group, immunofluorescence intensity of IL-1β and vimentin increased by 10.5-fold and 2.1-fold, respectively. Elevated IL-1β reflects inflammasome-mediated inflammatory signaling that promotes EMT initiation through TGF-β–dependent pathways, whereas increased vimentin expression represents acquisition of a mesenchymal phenotype, a hallmark of EMT and fibrotic remodeling. (Figure 9A,B). NAC treatment restored both markers to levels comparable to controls. Collectively, these findings demonstrate that NAC effectively suppresses inflammasome-mediated inflammatory signaling and mitigates EMT-associated fibrotic remodeling, thereby protecting renal tubular epithelial cells from high glucose- and lipid-induced injury.

### 3.6. NAC Interacts with G6Pase or PEPCK

**Figure 10:**
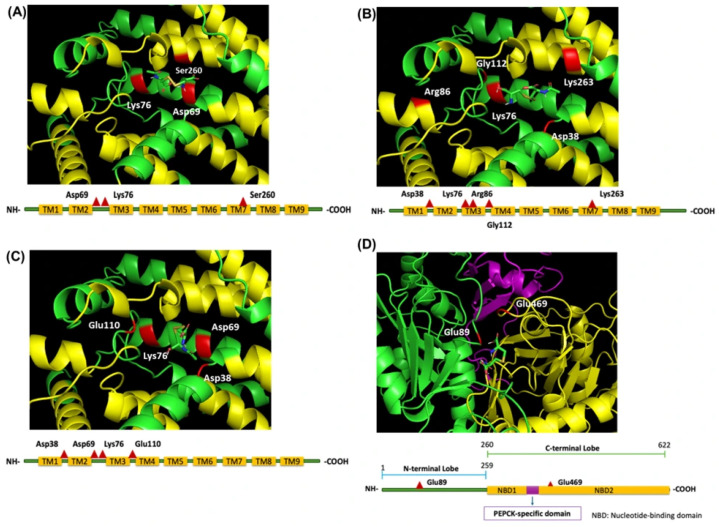
Molecular docking analysis of NAC with G6Pase and PEPCK. Predicted docking of detailed interaction diagrams showing NAC binding regions of G6Pase and PEPCK, involving interaction of NAC with G6Pase near residues (**A**) Asp69, Lys76, Ser260 (M0, binding affinity: −6.0); (**B**) Arg86, Gly112, Lys76, Lys263 (M1, Binding affinity: −5.5); (**C**) Asp38, Asp69, Lys76, Glu110 (M2, binding affinity: −5.3); and (**D**) Predicted docking interaction of NAC with PEPCK near residues Glu89 and Glu469 (M0, binding affinity: −5.1). The red triangles indicate the residues in protein structures, yellow boxes indicate the functional domains.

The molecular docking analyses indicated that NAC preferentially interacts with G6Pase, particularly within the M0–M2 docking regions involving residues associated with substrate recognition and catalytic regulation, including Asp69, Lys76, Arg86, Gly112, and Glu110 (Figure 10A–C). These regions are located near functional motifs important for phosphate binding and catalytic activity. The binding affinity between NAC and M0, M1, M2 in G6Pase is −6.0, −5.5 and −5.3. In Figure 10D, NAC binding to PEPCK displayed lower predicted affinity (−5.1) and more dispersed interaction sites, primarily near regulatory rather than catalytic regions.

## 4. Discussion

This study demonstrates that N-acetylcysteine (NAC) confers significant renoprotective effects in ApoE^−/−^ mice subjected to cardiometabolic stress induced by a high-fat diet and streptozotocin administration. Our findings reveal that NAC attenuates renal functional impairment and fibrotic remodeling by coordinately modulating inflammasome activation, insulin-associated PI3K/Akt–FOXO1 signaling, gluconeogenic reprogramming, and epithelial–mesenchymal transition (EMT). These results provide mechanistic insight into how cardiometabolic stress drives kidney disease progression and identify NAC as a modulator of metabolic–inflammatory crosstalk in the kidney.

Cardio-renal metabolic (CRM) syndrome represents a complex pathological state in which metabolic dysfunction [36,37,38], chronic inflammation [39,40,41], and cardiovascular abnormalities converge to accelerate renal injury [40]. In line with clinical observations, ApoE^−/−^ mice exposed to combined lipid and glycemic stress exhibited pronounced renal structural damage, inflammation, and fibrosis. During KD progression, inflammation serves as a critical pathogenic factor that contributes to tubular epithelial cell apoptosis, endothelial dysfunction, and tissue fibrosis [42,43]. Clinical studies have reported that oral administration of NAC replenishes glutathione depleted by disease processes, thereby exerting protective effects [34]. NAC enhances intracellular glutathione synthesis and additionally exhibits direct free radical-scavenging and anti-inflammatory properties [44]. Our results extend previous reports by demonstrating that these pathological changes are accompanied by marked activation of inflammasome signaling and dysregulation of renal gluconeogenesis, highlighting their coordinated contribution to CRM-associated kidney disease [40].

The current findings indicate that NAC attenuates KD progression in CRM syndrome by modulating inflammasome-associated pathways [45]. Upon activation, NLRP3 triggers caspase-1 activation, which subsequently cleaves and releases the mature proinflammatory cytokines IL-1β and IL-18, thereby driving sterile inflammation—a hallmark shared by type 2 diabetes, atherosclerosis, and kidney disease. In renal tubular epithelial cells, high glucose induced NLRP3 inflammasome activation, leading to enhanced caspase-1 activity and increased IL-1β secretion [46]. Similarly, in renal tubular cells affected by DKD, hyperglycemia and AGEs activate the NLRP3 inflammasome, leading to pyroptotic cell death and renal fibrosis. Consistent with these pathogenic mechanisms, our current experimental results demonstrate that NAC suppresses the expression of inflammasome-related proteins in the kidneys of ApoE^−/−^ mice. These findings suggest that NAC interferes with upstream inflammasome-triggering events, thereby limiting downstream inflammatory cascades that contribute to tubular injury and fibrosis.

Metabolic dysregulation and inflammation are tightly interconnected through insulin signaling pathways, with PI3K/Akt acting as a critical regulatory hub [47]. PI3K/AKT is central to insulin signaling and also regulates cell survival, proliferation, and metabolic homeostasis. Previous studies have shown that inflammatory cytokines such as TNF-α impair the phosphorylation of IRS, thereby suppressing PI3K/AKT activity and contributing to insulin resistance [43,48]. Conversely, activated AKT phosphorylates and inhibits the transcription factor FOXO1, preventing its nuclear translocation and subsequently reducing the expression of gluconeogenic genes, such as PEPCK and G6Pase [49,50]. Our results indicate that NAC enhances the phosphorylation of PI3K/AKT and inhibits FOXO1 nuclear localization. The renoprotective effects of NAC on the cardio–renal axis are likely mediated through both indirect and direct mechanisms. Specifically, NAC may indirectly suppress renal gluconeogenesis via modulating upstream inflammatory responses and the IRS-1/PI3K/Akt signaling pathway. In parallel, docking simulations suggest a potential for direct modulation of gluconeogenic enzyme activity. Molecular docking simulations indicate that NAC can interact with key gluconeogenic enzymes, including G6Pase and PEPCK. Specifically, NAC targets the G6Pase M0 region (Asp69, Lys76, Ser260) and distinct PEPCK domains. Notably, these predictive interactions lack direct validation via X-ray crystallography, representing a current study limitation. However, docking approaches were not accounted for dynamic conformational changes under physiological conditions, and the predicted binding affinities are relatively modest. Collectively, these findings support the notion that NAC predominantly regulates gluconeogenic reprogramming through normalization of the upstream signaling pathway rather than through direct inhibition of individual metabolic enzymes. Nevertheless, a limitation of the present study is the absence of direct functional assessment of renal gluconeogenic flux. Although NAC significantly reduced the expression of key gluconeogenic enzymes, including PEPCK and G6Pase, at the mRNA and/or protein levels, these molecular changes do not directly demonstrate a reduction in renal glucose production. Functional assays measuring glucose output from gluconeogenic substrates, such as pyruvate, lactate, or glycerol, as well as isotope-labeled tracer analyses, would provide more definitive evidence regarding the effect of NAC on renal gluconeogenic flux. Therefore, the present findings should be interpreted as evidence that NAC modulates gluconeogenesis-related molecular signaling, while future studies are required to confirm its functional impact on renal glucose production. Furthermore, the site-directed mutational analyses are also warranted to clarify the relative contributions of these mechanisms to the protective effects of NAC against CRM injury.

EMT represents a pivotal mechanism linking chronic inflammation and metabolic stress to renal fibrosis [51]. Consistent with this paradigm, cardiometabolic stress induced pronounced EMT, characterized by loss of epithelial markers and induction of mesenchymal and profibrotic proteins [52]. In this study, NAC treatment effectively reversed these EMT-associated alterations, suggesting that suppression of inflammasome activation and restoration of metabolic signaling collectively contribute to preservation of epithelial integrity. Notably, emerging evidence indicates that metabolic reprogramming itself influences EMT progression [53,54,55], and our findings support the concept that normalization of gluconeogenic activity can interrupt the feed-forward cycle of metabolic stress, inflammation, and fibrotic remodeling.

This study delineates key mechanisms underlying NAC-mediated renoprotection in CRM syndrome; several limitations warrant consideration. First, in the present study, the cardiac tissue analysis was not included, although cardio-renal metabolic syndrome involves pathological interactions between the heart, kidney, and metabolic systems. Therefore, the current findings primarily support the renoprotective effects of NAC under cardiometabolic stress, rather than direct cardioprotective effects. Future studies incorporating parallel cardiac and renal tissue analyses are warranted to clarify the impact of NAC on heart–kidney crosstalk and systemic cardio-renal remodeling. Second, the use of young ApoE^−/−^ mice may not fully recapitulate age-associated metabolic dysfunction observed in human disease, and future studies employing aged models are needed to enhance translational relevance [56,57,58]. Third, this study focused primarily on tubular pathology, and the effects of NAC on glomerular components, including podocytes and endothelial cells, remain to be explored [59,60]. Fourth, NAC was administered as a preventive intervention; whether treatment efficacy is observed in established CRM-associated kidney disease requires further investigation [61]. Fifth, a single standard dose (200 mg/kg) was used in the in vivo experiments without the active comparators like SGLT2 inhibitors. Although this dose was selected based on previous experimental studies, the lack of a dose–response analysis prevents the determination of the minimal effective dose, optimal therapeutic range, and dose-dependent efficacy of NAC. Sixth, lacking direct gluconeogenic flux analysis, future isotope tracing is needed to confirm the functional impact of NAC on renal gluconeogenic capacity. Seventh, although the affordability and high safety profile of NAC enhance its therapeutic feasibility in CKD, its clinical translation faces significant challenges in advanced patients. These include altered pharmacokinetics, optimal dosing uncertainties, and a critical need for large-scale randomized controlled trials to confirm long-term safety [32,62,63,64].

## 5. Conclusions

In summary, this study identifies aberrant renal gluconeogenesis and inflammasome activation as interconnected pathogenic drivers of fibrosis under cardiometabolic stress and demonstrates that NAC mitigates kidney injury by modulating these pathways. By restoring metabolic signaling, suppressing inflammatory activation, and limiting EMT progression, NAC emerges as a mechanistic regulator of renal fibrosis rather than a nonspecific antioxidant. These findings provide a conceptual framework for targeting metabolic–inflammatory signaling networks in the treatment of kidney disease associated with cardiometabolic disorders.

## Figures and Tables

**Table 1:** Effects of NAC in ApoE^−/−^ mice exposed to STZ+HFD.

	C	NC	STZ	STZ+HFD	STZ+HFD+NAC ^1,2^
BUN (mg/dL)	23.73 ± 4.35 ^a^	26.34 ± 3.13 ^a^	30.27 ± 2.06 ^b^	40.79 ± 5.66 ^b^	21.02 ± 3.51 ^a^
CRE (mg/dL)	0.40 ± 027 ^a^	0.43 ± 0.15 ^a^	0.52 ± 0.1 ^b^	1.18 ± 0.40 ^c^	0.55 ± 0.08 ^b^
UA (mg/dL)	4.75 ± 0.85 ^a^	5.71 ± 1.13 ^a^	7.90 ± 0.57 ^b^	8.12 ± 0.95 ^b^	6.27 ± 0.73 ^c^
HbA1c (%)	4.08 ± 0.13 ^a^	4.04 ± 0.32 ^a^	5.73 ± 1.69 ^b^	6.61 ± 0.72 ^b^	4.95 ± 1.00 ^c^
Insulin (pg/L)	0.295 ± 0.016 ^a^	0.267 ± 0.010 ^a^	0.338 ± 0.024 ^b^	0.348 ± 0.019 ^b^	0.297 ± 0.011 ^a^

^1^ ApoE^−/−^ mice were treated with intraperitoneal-injected STZ at a dose of 50 mg/kg body weight and HFD, subsequently supplemented with 200 mg/kg body weight NAC for 8 weeks. The C, NC (ApoE^−/−^ mice) and STZ groups were given the standard chow diet. ^2^ Each value is expressed as mean ± SE (*n* = 5). Results were statistically analyzed using ANOVA. Values not sharing a common letter in the same row are significantly different (*p* < 0.05).

## Data Availability

The original contributions presented in this study are included in the article. Further inquiries can be directed to the corresponding authors.
